# Integration of metabolomics, transcriptomics, and microRNA expression profiling reveals a miR-143-HK2-glucose network underlying zinc-deficiency-associated esophageal neoplasia

**DOI:** 10.18632/oncotarget.18434

**Published:** 2017-06-09

**Authors:** Louise Y. Fong, Ruiyan Jing, Karl J. Smalley, Cristian Taccioli, Johannes Fahrmann, Dinesh K. Barupal, Hansjuerg Alder, John L. Farber, Oliver Fiehn, Carlo M. Croce

**Affiliations:** ^1^ Department of Pathology, Anatomy & Cell Biology, Thomas Jefferson University, Philadelphia, PA, USA; ^2^ Sidney Kimmel Cancer Center, Thomas Jefferson University, Philadelphia, PA, USA; ^3^ Center for Molecular Carcinogenesis, Thomas Jefferson University, Philadelphia, PA, USA; ^4^ Animal Medicine, Production and Health Department, University of Padua, Padua, Italy; ^5^ University of California, Davis, West Coast Metabolomics Center, Davis, CA, USA; ^6^ Department of Biochemistry, Faculty of Sciences, King Abdulaziz University, Jeddah, Saudi Arabia; ^7^ Department of Molecular Virology, Immunology, and Medical Genetics, Comprehensive Cancer Center, The Ohio State University, Columbus, OH, USA

**Keywords:** metabolomic profiling, transcriptomics and microRNA profiling integration, esophageal neoplasia, dietary zinc-deficiency, miR-143 - Hk2 - glucose signaling

## Abstract

Esophageal squamous cell carcinoma (ESCC) in humans is a deadly disease associated with dietary zinc (Zn)-deficiency. In the rat esophagus, Zn-deficiency induces cell proliferation, alters mRNA and microRNA gene expression, and promotes ESCC. We investigated whether Zn-deficiency alters cell metabolism by evaluating metabolomic profiles of esophageal epithelia from Zn-deficient and replenished rats *vs* sufficient rats, using untargeted gas chromatography time-of-flight mass spectrometry (*n* = 8/group). The Zn-deficient proliferative esophagus exhibits a distinct metabolic profile with glucose down 153-fold and lactic acid up 1.7-fold (*P* < 0.0001), indicating aerobic glycolysis (the “Warburg effect”), a hallmark of cancer cells. Zn-replenishment rapidly increases glucose content, restores deregulated metabolites to control levels, and reverses the hyperplastic phenotype. Integration of metabolomics and our reported transcriptomic data for this tissue unveils a link between glucose down-regulation and overexpression of HK2, an enzyme that catalyzes the first step of glycolysis and is overexpressed in cancer cells. Searching our published microRNA profile, we find that the tumor-suppressor miR-143, a negative regulator of HK2, is down-regulated in Zn-deficient esophagus. Using *in situ* hybridization and immunohistochemical analysis, the inverse correlation between miR-143 down-regulation and HK2 overexpression is documented in hyperplastic Zn-deficient esophagus, archived ESCC-bearing Zn-deficient esophagus, and human ESCC tissues. Thus, to sustain uncontrolled cell proliferation, Zn-deficiency reprograms glucose metabolism by modulating expression of miR-143 and its target HK2. Our work provides new insight into critical roles of Zn in ESCC development and prevention.

## INTRODUCTION

Although cancer mortality rates have declined worldwide since the mid 1980s, esophageal squamous cell carcinoma (ESCC), the predominant esophageal cancer subtype, has a 5-year survival of only 10% [1]. Due to lack of early symptoms, ESCC is typically diagnosed at an advanced stage that defies treatment by surgery combined with radio/chemotherapy. Thus, clarification of the mechanisms underlying the pathogenesis of ESCC and development of new prevention and therapeutic strategies are critically needed.

Zinc (Zn)-deficiency (ZD) is recognized as a major worldwide public health problem [2-6], affecting ∼31% of the global population with higher rates in developing countries [5]. Although severe ZD is uncommon, mild-to-moderate ZD is prevalent throughout the world [7]. Epidemiologic and clinical studies have implicated dietary ZD in the etiology of ESCC [8-10]. Specifically, Abnet et al. [11] demonstrated that tissue Zn concentration is inversely associated with the subsequent risk of developing ESCC. Because Zn is required for the activity of hundreds of enzymes, for proper immune function, and for the conformation of > 2000 transcription factors that control cell proliferation, apoptosis, and signaling pathways [12, 13], ZD predisposes to disease by adversely affecting many processes.

Cell proliferation is a hall mark of cancer [14]. Previously, we showed that rats on a ZD diet for ∼6 weeks develop a hyperplastic esophagus with sustained, uncontrolled cell proliferation [15]. In transcriptome analyses using DNA microarrays, the ZD esophagus showed a distinct gene expression signature with the proinflammation mediators *S100a8/a9* as the top up-regulated genes [16]. Zn-replenishment (ZR) rapidly restored to control levels the expression of S100A8/A9 and other genes and reversed the hyperplastic phenotype [16]. In addition, prolonged ZD (21 weeks) leads to an expanded cancer-associated inflammatory program that when combined with non-carcinogenic low doses of the environmental carcinogen *N*-nitrosomethylbenzylamine (NMBA), elicited a 67% incidence of ESCC [17]. Among the up-regulated genes were genes known as hallmarks of cancer-related inflammation (chemokines, cytokines, prostaglandins) implicated in the development of human cancers, including ESCC [18, 19]. Again, ZR reversed this inflammatory gene signature and prevented cancer formation [17, 20]. Thus, our ZD rat esophageal cancer model reproduces the path to human ESCC and provides an opportunity to understand the molecular basis of ESCC associated with ZD.

MicroRNAs (miRNA) are short non-coding RNAs that regulate gene expression by translational inhibition and mRNA degradation. Individual miRNAs can inhibit multiple target genes or entire signaling pathways, including cell proliferation, differentiation, and apoptosis. miRNA expression levels are altered in all human cancers studied, including ESCC [21], can act as oncogenes or tumor suppressors [22, 23] and have emerged as therapeutic targets for cancer [24]. Using the NanoString microRNA expression profiling platform, we showed that ZD promotes ESCC by inducing an oncogenic miRNA signature that resembles the human ESCC miRNAome [25] with up-regulation of oncogenic miR-31, -223, and -21 [26-28]. Additionally, ESCC development and the underlying miRNA dysregulation are dependent on the extent of dietary deficiency of Zn [28].

In the early 1920s, Warburg discovered that unlike normal tissues, tumor cells convert glucose to lactate even under aerobic condition, establishing that cancer cells exhibit altered cellular metabolism fundamental to carcinogenesis [29, 30]. In the ensuing years, this phenomenon of aerobic glycolysis or Warburg effect has been documented in a variety of human cancers, including ESCC [31], most readily by assessing glucose uptake using positron emission tomography (PET) with a radiolabeled analog of glucose (^118^F-fuorodeoxyglucose, FDG) as reporter. Altered metabolism is now considered a hallmark of cancer [14], and deregulated uptake of glucose is a common feature of cancer-associated metabolic changes [32].

Given that altered metabolism results from active reprogramming by altered oncogenes and tumor suppressors [33], these metabolite changes are downstream and complementary to changes in genes and proteins and are key players in biological networks. Additionally, miRNAs have emerged as key regulators of cellular metabolism in normal and pathological conditions [34]. For example, miR-122 and its host gene regulate cholesterol and lipid metabolism in liver [35]; miR-143 and its target gene, HK2, regulates aerobic glycolysis in tumor cells [36-39].

We hypothesized that in addition to inducing a hyperplastic esophageal phenotype along with oncogenic alterations in mRNA and miRNA gene expression, dietary ZD also causes altered cellular metabolism during early esophageal carcinogenesis. To investigate this, we performed untargeted metabolomic profiling by gas chromatography time-of-flight mass spectrometry (GC-TOF MS) to evaluate metabolite changes in esophageal mucosa obtained from ZD rats *vs* ZS rats [16]. GC-TOF MS based metabolic profiling has been used for studying primary metabolism (sugars, lipids, & small peptides) in the characterization of human cancers such as ovarian carcinoma [40, 41] and lung adenocarcinoma [42-44]. To gain insights into the molecular basis of alterations in biochemical pathways, we integrated metabolomics of ZD rat esophagus with our reported transcriptome dataset for this tissue from a study of identical design [16]. We also investigated whether a miRNA-mRNA-metabolite network can be established in early ZD-associated esophageal tumor development by searching our reported miRNA profile data [28].

## RESULTS

### Esophageal mucosa samples for GC-TOF MS-based analysis

Esophageal mucosa samples were obtained from Zn-modulated rats after a 6-week dietary regimen as described before [16]. Consistent with previous studies [15, 16, 20], the Zn status marker, testis Zn content (µg/g dry weight, mean ± SD), was significantly lower in ZD than control ZS rats (105 ± 18 *vs* 131 ± 6.8, *P* < 0.01, *n* = 8 rats/group). Also, the ZD esophagus was hyperplastic with a high rate of cell proliferation, as compared with ZS esophagus that is typically 2 to 3 cells thick. Seventy-two hours after replenishing Zn, testis zinc content in ZR rats was 139 ± 11 µg/g dry weight, a result comparable to ZS rats. At the same time, ZR esophagus reverted to a near normal esophageal phenotype, consistent with previous studies [16, 20].

### Metabolic profile of hyperplastic ZD esophagus

**Figure 1:**
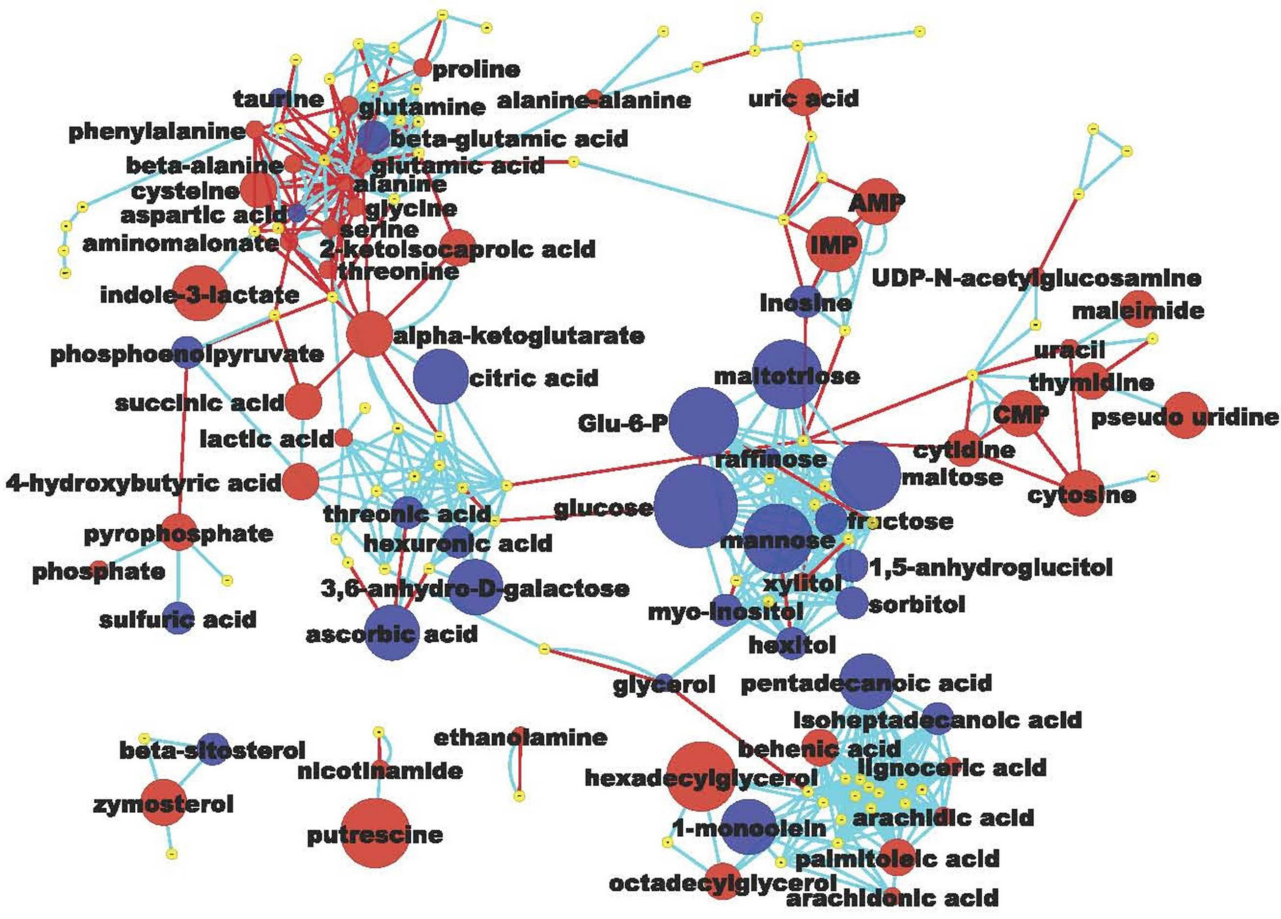
Metabolomic network of biochemical differences between hyperplastic Zn-deficient and Zn-sufficient esophagus Each node represents an identified metabolite. Metabolites are connected based on biochemical relationships (red, KEGG RPAIRS) or structural similarity (light blue, Tanimoto coefficient ≥ 0.7). Metabolite size reflects fold-change (ZD *vs* ZS); node color represents relative change (Blue, decrease, *P* < 0.05; Red, increase, *P* < 0.05; yellow, insignificant change) metabolite size reflects absolute fold change (Table 1). Labels are shown for the metabolites that pass the significance cutoff (*P* < 0.05)

Metabolomic analysis by GC-TOF MS in esophageal mucosa from ZD and ZS rats (*n* = 8 per group) yielded a total of 353 compounds, of these 155 were structurally known metabolites and 198 were unknown compounds. Using a cutoff point of *P* value < 0.05 and ≥ 1.3-fold difference, the proliferative ZD esophagus exhibited a distinct metabolic signature as compared with its non-proliferative ZS counterpart. A total of 71 known metabolites were significantly altered, with 27 metabolites down-regulated and 44 up-regulated (Table 1). To display the biochemical differences between hyperplastic ZD and control ZS esophagus, a metabolomic network [45] was calculated among the structurally identified metabolites using the Kyoto encyclopedia of genes and genomes (KEGG ) databases [46] and PubChem CIDs identifiers [47] (Figure 1)*.*

**Table 1:** Metabolomic signature in precancerous Zn-deficient rat esophagus

Metabolite name	Fold change (ZD *vs* ZS)	*P*-value	Biological process
27 down-regulated			
glucose	-153	0.0002	Glycolysis/gluconeogenesis
maltose	-20	0.0002	Starch & sucrose metabolism
maltotriose	-9.0	0.0002	ATP-binding cassette transporters
glucose-6-phosphate	-8.8	0.0011	Glycolysis/pentose phosphate pathway
mannose	-7.5	0.0011	Fructose & mannose metabolism
3,6-anhydro-D-galactose	-6.7	0.0100	---
ascorbic acid	-6.1	0.0009	Glutathione metabolism
1-monoolein	-5.9	0.0002	---
citric acid	-5.3	0.0003	Citrate cycle (TCA Cycle)
pentadecanoic acid	-5.1	0.0002	---
hexitol	-3.6	0.0002	Fructose & mannose metabolism
beta-glutamic acid	-3.4	0.0022	---
fructose	-3.4	0.0002	Starch & sucrose metabolism
phosphoenolpyruvate	-3.3	0.0017	Glycolysis/gluconeogenesis
myo-inositol	-2.9	0.0001	Galactose Metabolism
sulfuric acid	-2.9	0.0012	Purine metabolism
isoheptadecanoic acid NIST	-2.9	0.0150	---
beta-sitosterol	-2.8	0.0001	Steroid biosynthesis
threonic acid	-2.4	0.0018	Ascorbate & aldarate metabolism
inosine	-2.3	0.0370	Purine metabolism
1,5-anhydroglucitol	-2.2	0.0002	---
sorbitol	-2.1	0.0019	Fructose & mannose metabolism
hexuronic acid	-2.0	0.0003	---
raffinose	-1.9	0.0027	Galactose metabolism
aspartic acid	-1.8	0.0415	Amino acid metabolism
taurine	-1.7	0.0019	Primary bile acid metabolism
glycerol	-1.4	0.0085	Galactose/glycerolipid metabolism
44 up-regulated			
putrescine	14	0.0100	Glutathione/amino acid metabolism
hexadecylglycerol NIST	10	0.0002	---
inosine 5'-monophosphate	6.4	0.0063	Purine metabolism
indole-3-lactate	5.5	0.0140	Tryptophan degradation
cytidine-5-monophosphate NIST	4.7	0.0013	Pyrimidine metabolism
zymosterol	3.7	0.0000	---
alpha-ketoglutarate	3.5	0.0380	---
cytosine	3.3	0.0002	Pyrimidine metabolism
adenosine-5-monophosphate	3.0	0.0011	Purine metabolism
pseudo uridine	3.0	0.0100	Pyrimidine metabolism
cytidine	2.6	0.0003	Pyrimidine metabolism
cysteine	2.4	0.0000	Amino acid metabolism
octadecylglycerol	2.4	0.0030	---
uric acid	2.3	0.0100	Purine metabolism
succinic acid	2.2	0.0070	Citrate cycle (TCA cycle)
thymidine	2.1	0.0047	Pyrimidine metabolism
maleimide	2.1	0.0067	---
2-ketoisocaproic acid	2.1	0.0149	Amino acid metabolism
palmitoleic acid	2.1	0.0030	Fatty acid biosynthesis
4-hydroxybutyric acid	2.1	0.0000	Butanoate metabolism
pyrophosphate	2.0	0.0043	Oxidative phosphorylation
behenic acid	2.0	0.0002	Biosynthesis of unsaturated fatty acids
aminomalonate	1.8	0.0100	---
beta-alanine	1.8	0.0402	Pyrimidine metabolism
UDP-N-acetylglucosamine	1.8	0.0047	Amino sugar & nucleotide sugar metabolism
arachidic acid	1.7	0.0001	Biosynthesis of unsaturated fatty acids
lactic acid	1.7	0.0000	Glycolysis, pyruvate metabolism
alanine-alanine	1.7	0.0011	D-alanine metabolism
ethanolamine	1.7	0.0001	---
xylitol	1.6	0.0380	Riboflavin metabolism
lignoceric acid	1.6	0.0044	Biosynthesis of unsaturated fatty acids
phosphate	1.6	0.0037	Oxidative phosphorylation
arachidonic acid	1.6	0.0150	Biosynthesis of unsaturated fatty acids
proline	1.5	0.0124	Amino acid metabolism
phosphoethanolamine	1.5	0.0438	Glyerophospholipid metabolism
uracil	1.5	0.0395	Pyrimidine metabolism
glutamine	1.4	0.0148	Amino acid metabolism C/N balance
phenylalanine	1.4	0.0132	Amino acid metabolism
serine	1.4	0.0000	Amino acid metabolism
alanine	1.3	0.030	Amino acid metabolism
glutamic acid	1.3	0.001	Amino acid metabolism
nicotinamide	1.3	0.021	Energy metabolism
threonine	1.3	0.009	Amino acid metabolism
glycine	1.3	0.001	Amino acid metabolism

A group of 71 significantly altered metabolites were identified in ZD vs ZS esophagus using a cutoff point of P-value ≤5% and fold-change ≥1.3 (n = 8 rats/cohort). ZD = Zn-deficient; ZS = Zn-sufficient.

Of the 27 metabolites that were significantly down-regulated in the hyperplastic ZD esophagus, sixteen (59%) were carbohydrates, including glucose, glucose-6-phosphate, maltose, maltotriose, mannose, 3,6-anhydro-D-galactose, and fructose. The remaining 41% were lipids (three), amino acids (three), nucleotide (one), sterol (one), organic acid (one), and others (two) (Figure 1 and Table 1). Most strikingly, glucose was decreased by 153-fold *(P* < 0.001*).* Glucose depletion was positively correlated with a 1.7-fold increase in lactic acid (*P <* 0.001)*.* This result indicates that the proliferating ZD-esophageal cells vigorously consume glucose (resulting in glucose depletion), and produce lactic acid under aerobic conditions.

Because of the need to replicate all of its cellular contents, proliferating cells have a high biosynthetic demand for amino acids, lipids, and nucleotides. Thus, in cancer cells aerobic glycolysis fulfills the requirement to support cell proliferation rather than to generate energy in the form of adenosine 5’-triphosphate (ATP) [48]. The highly proliferative ZD esophagus showed a metabolic profile consistent with uncontrolled proliferation. Of the 44 up-regulated metabolites (Table 1 and Figure 1), many were involved in amino acid metabolism/protein biosynthesis (putrescine, cysteine, beta-alanine, ethanolamine, proline, glutamine, phenylalanine, nicotinamide etc.), purine & pyrimidine metabolism (inosine 5’-monophosphate, cytidine-5-monophosphate, adenosine-5-monophosphate, cytidine, uric acid, thymidine, phosphate), unsaturated fatty acids biosynthesis (hexadecylglycerol, octadecylglycerol, arachidonic acid, palmitoleic acid, arachidic acid), glycolysis (lactic acid), and energy metabolism (nicotinamide). Of these, the top up-regulated metabolite was the amino-acid derived putrescine (up 14-fold, *P <* 0.01)*.* High levels of polyamines, including putrescine, are associated with human cancers and cell proliferation through DNA packaging [49, 50]. In summary, untargeted metabolomic profiling by GC-TOF MS revealed that the proliferative ZD esophageal mucosa exhibits extensive remodeling of metabolism, including a high rate of aerobic glycolysis accompanied by up-regulation of many metabolites that serve as building blocks for biomass synthesis.

### Reversal of cancer-associated metabolic profile by ZR

To investigate the metabolic response following Zn replenishment, we performed metabolomic analysis by GC-TOF MS in restored esophageal mucosa from ZR rats at 72 hours after replenishing Zn (*n* = 8 rats). As shown Table 2, the number of significantly deregulated metabolites in ZR *vs* ZS esophagus was 30 (13 down-regulated & 17 up-regulated), which is ∼42% of that in ZD vs ZS esophagus (Table 1). In particular, glucose deregulation was improved from a down-regulation of 153-fold in ZD esophagus to 20-fold in ZR counterpart (*P <* 0.001, Table 2), as compared to ZS esophagus. Putrescine, the top-up-regulated metabolite in ZD esophagus was returned to ZS status. These metabolomic results mirror those of transcriptomic data, in which, shortly after ZR the altered gene expression rapidly reverted to near control ZS levels, accompanied by reduced cell proliferation, increased apoptosis, and a reversal of the hyperplastic esophageal phenotype [16, 17, 20].

**Table 2:** Metabolomic profiling of esophageal mucosa from Zn-replenished rats at 72 hours after Zn-replenishment as compared to Zn-sufficient counterpart

Metabolite name	Fold change (ZR *vs* ZS)	*P*-value	Biological process
13 down-regulated			
glucose	-20	0.0006	Glycolysis/gluconeogenesis
pentadecanoic acid	-5.8	0.0002	---
3,6-anhydro-D-galactose	-3.7	0.0100	ATP-binding cassette transporters
maltose	-3.1	0.0104	Starch & sucrose metabolism
sorbitol	-3.0	0.0002	Fructose & mannose metabolism
linoleic acid	-2.2	0.0171	Biosynthesis of prostaglandin
beta-glutamic acid	-1.9	0.0371	---
isoheptadecanoic acid NIST	-1.9	0.0210	---
fumaric acid	-1.7	0.0100	Citrate cycle (TCA cycle)
hexuronic acid	-1.6	0.0135	---
1,5-anhydroglucitol	-1.5	0.0047	---
beta-sitosterol	-1.5	0.0361	Steroid biosynthesis
N-acetylmannosamine	-1.4	0.0348	Amino/nucleotide sugar metabolism
17 up-regulated			
indole-3-lactate	2.8	0.0031	Tryptophan degradation
ornithine	2.2	0.0463	Arginine biosynthesis
tyrosine	2.0	0.0003	Amino acid metabolism
proline	1.7	0.0010	Amino acid metabolism
aminomalonate	1.7	0.0100	---
citrulline	1.7	0.0011	Arginine biosynthesis
alanine-alanine	1.7	0.0011	D-alanine metabolism
leucine	1.7	0.0004	Amino acid metabolism
oxoproline	1.6	0.0002	---
phenylalanine	1.6	0.0005	Amino acid metabolism
isoleucine	1.6	0.0002	Amino acid metabolism
valine	1.6	0.0002	Amino acid metabolism
methionine sulfoxide	1.5	0.0054	---
trans-4-hydroxyproline	1.5	0.0108	Amino acid metabolism
glutamine	1.5	0.0047	Amino acid metabolism C/N balance
glycine	1.4	0.0000	Amino acid metabolism
threonine	1.4	0.0023	Amino acid metabolism

A group of 30 significantly altered metabolites were identified in ZR vs ZS esophagus using a cutoff point of P-value ≤5% and fold-change ≥1.3 (n = 8 rats/cohort). ZR = Zn-replenished; ZS = Zn-sufficient

### Integration of metabolomics, transcriptomics, and miRNA profiling dataset

To identify a key deregulated biochemical and molecular signaling network in ZD-induced esophageal preneoplasia, we integrated metabolomics with transcriptomics of ZD esophagus from a study of identical design [16]. Previous microarray analysis by Affymetrix rat genome 230 2.0 GeneChip identified a total of 2305 significantly deregulated genes in the hyperplastic ZD *vs* ZS esophagus (fold change ≥ 2) from rats after a 6-week dietary regimen [16]. A gene-metabolite integration network (Supplementary Figure 1) was calculated by queries in ZD *vs* ZS esophagus from the 2,305 deregulated genes [16] and 71 significantly deregulated metabolites (Table 1) and mapping with log2 fold-change using the GRINN database within the metabox toolbox [51]. Several metabolic pathways showed significant alterations at both the transcriptional and metabolic levels (Supplementary Figure 1). As examples, connections that occur between specific gene and metabolite were observed between *Hk2* up-regulation & glucose down-regulation; cytochrome P450 genes (*Cyp4b1*, *Cyp4f6*, *Cyp2a1*) down-regulation & arachidonic acid up-regulation; *Atp2a2*, *Atp1a2* down-regulation/*Atp7a, Dusp1, Pfkfb3* up-regulation & phosphate (O_4_P^-3^) up-regulation; *Gfpt2* down-regulation & L-glutamine/L-glutamic acid up-regulation.

**Figure 2:**
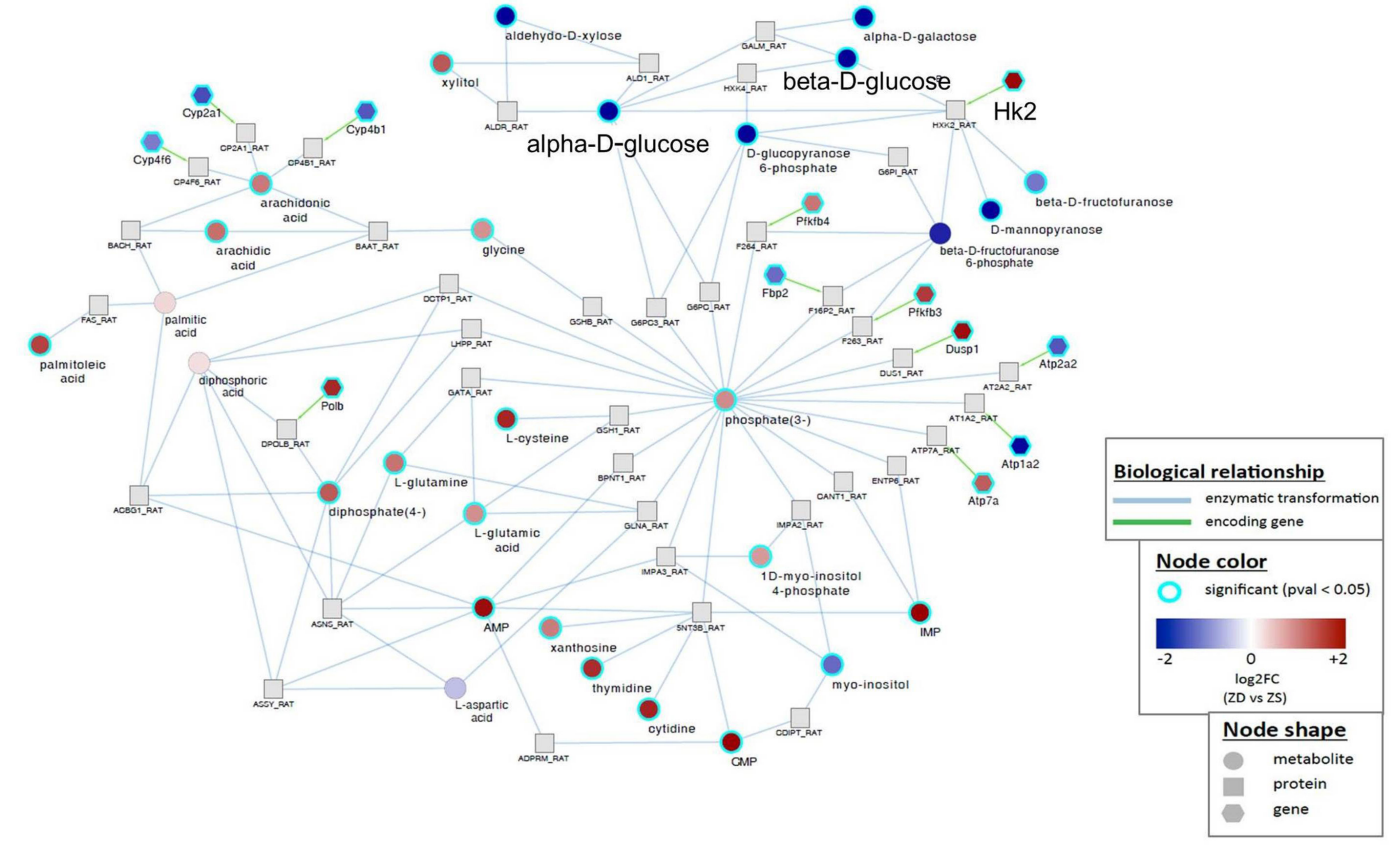
Gene-metabolite integration network in Zn-deficient *versus* Zn-sufficient esophagus reveals a direct connection between *Hk2* up-regulation and decreased level of glucose metabolite Biological relationship - blue, enzymatic transformation; green, encoding gene. Node shape represents metabolite, protein, or gene. Node color represents fold change (ZD *vs* ZS) and *P* < 0.05.

We focused our study on HK2-glucose link because of its critical role in cancer metabolism [32]. Strikingly, *HK2* that was up-regulated 3.9-fold in hyperplastic ZD *vs* ZS esophagus by microarray expression analysis [16] was directly connected to down-regulated glucose metabolites, namely, alpha-D-glucose and beta-D-glucose (Figure 2). The HK2 enzyme catalyzes the first committed step in glucose metabolism where glucose is phosphorylated to yield glucose-6-phosphate. In mammals there are four hexokinase isoforms, HK1, HK2, HK3, and HK4 (also known as glucokinase). Only HK2 is expressed at high levels in cancer cells [52, 53], thus accounting for the high glycolytic rate in cancer cells [54]. Thus, by combining metabolomics and transcriptomics, we have identified an aerobic glycolysis network that involves HK2 and glucose signaling associated with uncontrolled cell proliferation in early esophageal cancer development induced by dietary ZD.

**Figure 3:**
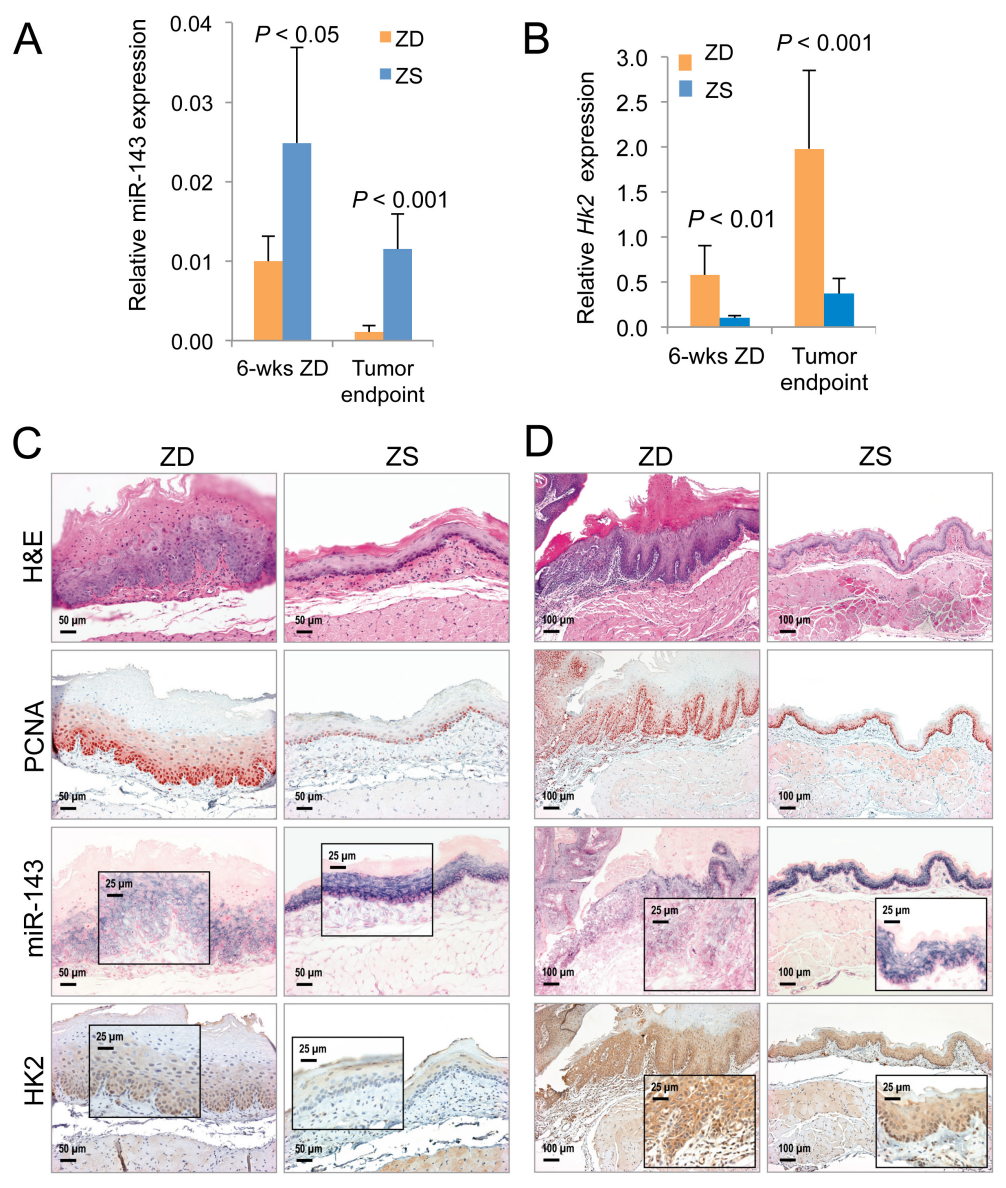
Analysis of cell proliferation, miR-143 and HK2 expression in Zn-deficient preneoplastic and neoplastic rat esophageal tissues **A.** qPCR analysis of miR-143 (U87 as normalizer, *n* = 8 rats/group; results are shown as means, error bars represent standard deviation). **B.** qPCR analysis of mRNA expression of *HK2* (Psmb6 as normalizer, *n* = 8 rats/group; results are shown as means, error bars represent standard deviation). Analysis of miR-143 and HK2 expression by *in situ* hybridization (ISH) and immunohistochemistry (IHC) in formalin fixed paraffin embedded esophageal samples in - **C.** ZD *versus* ZS esophagus after a 6-week dietary regimen (*n* = 8 rats/group) **D.** Archived samples of ESCC-bearing ZD esophagus *versus* tumor-free control ZS esophagus [28] (*n* = 10 rats/group). Representative hematoxylin and eosin [H&E]-stained and PCNA-stained sections are shown. miR-143 ISH signal (blue, 4-nitro-blue tetrazolium and 5-brom-4-chloro-3′-indolylphosphate; counterstain, nuclear fast red). HK2 expression (brown, 3,3′-diaminobenzidine tetrahydrochloride, counterstain, Harris modified hematoxylin). Scale bars: 50 μm (x200 magnification), 100 μm (x100 magnification, and 25 μm (x400 magnification, inset).

Using the NanoString miRNA expression profiling platform, we previously reported that prolonged ZD (22 weeks) induced extensive alterations in miRNA expression in the hyperplastic ZD esophagus, and many of the dysregulated miRNAs are similarly altered in human ESCC [28]. In a search for known miRNAs that regulate HK2, we examined this published miRNA profiling data for ZD esophagus [28]. Significantly, miR-143, a tumor suppressor in human cancers, including ESCC [55, 56], and a known negative regulator of *HK2* [36], was down-regulated 1.8-fold (*P =* 0.0136) in the highly proliferative ZD esophagus as compared to its non-proliferative counterpart *vs* ZS [28]. Based on this observation, we went on to perform Taqman miRNA assays using qPCR and showed that the tumor suppressor miR-143 was down-regulated 2.5-fold in hyperplastic ZD *vs* ZS esophagus from rats after a brief 6-week dietary regimen (*P* < 0.05, *n* = 8 rats/group*)*, and down-regulated 10-fold (*P* < 0.001*, n* = 8 rats/group) in archived samples of ESCC-bearing ZD esophagus *vs* non-ESCC-bearing ZS counterpart from an esophageal tumor study in Zn-modulated rats [28]. In an inverse manner, qPCR analysis showed that *Hk2* mRNA levels were significantly up-regulated in these same ZD tissues (up 5.8-fold in hyperplastic ZD *vs* ZS esophagus & up 5.4-fold in archived samples [28] of ESCC-bearing ZD esophagus *vs* non-ESCC-bearing ZS esophagus (*P* < 0.01 to *P* < 0.001, *n* = 8 rats/group) (Figure 3B). Thus, integration of metabolomics with transcriptomics [16] & microRNA profiling data [28] in the hyperplastic ZD esophagus revealed that deregulated glucose uptake is accompanied by miR-143 down-regulation and up-regulation of the hexokinase gene *Hk2*. This result is consistent with reports that miR-143 is an essential regulator of cancer glycolysis by targeting HK2 in cancer cells, including lung cancer, head and neck squamous cell carcinoma, renal cell carcinoma, and colon cancer cells [36-39].

### Divergent inverse correlation of miR-143 & HK2 expression in nonproliferative esophagus *vs* proliferative ZD esophageal neoplasia and human ESCC

To understand the distribution and localization of miR-143 in esophageal neoplasia in relation to localization of its target HK2 protein and the level of cell proliferation, we performed *in situ* hybridization (ISH) and immunohistochemical staining (IHC) on near serial sections of rat esophageal tissues (*n* = 10 rats/group), as well as in the archived human ESCC tissues for which we previously reported overexpression of miR-31, -21, -223 [27, 28]. Cellular origin of miR-143 was defined by ISH using high affinity double Dig-labeled LNA probes (Exiqon, Vedbaek, Denmark). Localization of HK2 protein and the cell proliferation marker PCNA [56] were evaluated by IHC.

Both nonproliferative ZS esophagus (after a 6-week dietary regimen) and non-ESCC-bearing ZS rat esophagus (from an esophageal tumor study) showed few PCNA-positive nuclei, mostly restricted to basal cell layer (Figure 3C and 3D). Concurrently, these ZS tissues showed abundant and intense miR-143 ISH signal in basal and suprabasal cell layers along with weak HK2 protein expression. This finding indicates that robust expression of this miRNA suppresses HK2 expression, effects that support normal cell growth. By contrast, the highly proliferative preneoplastic ZD esophagus, as well as ESCC-bearing ZD esophagus displayed a high rate of cell proliferation with abundant PCNA-positive nuclei in many cell layers. At the same time, both preneoplastic and cancerous phenotypes exhibited weak/diffuse miR-143 ISH signals but moderate/strong cytoplasmic HK2 expression (Figure 3C and 3D), indicating down-regulation of the tumor suppressor, miR-143, leads to overexpression of HK2 and enhanced cell proliferation/ESCC development. Together, these data revealed for the first time the opposing inverse-correlation between miR-143 & HK2 expression in normal esophagus with limited cell growth (ZS esophagus) and precancerous/cancerous ZD esophageal cells with unbridled cell proliferation.

**Figure 4:**
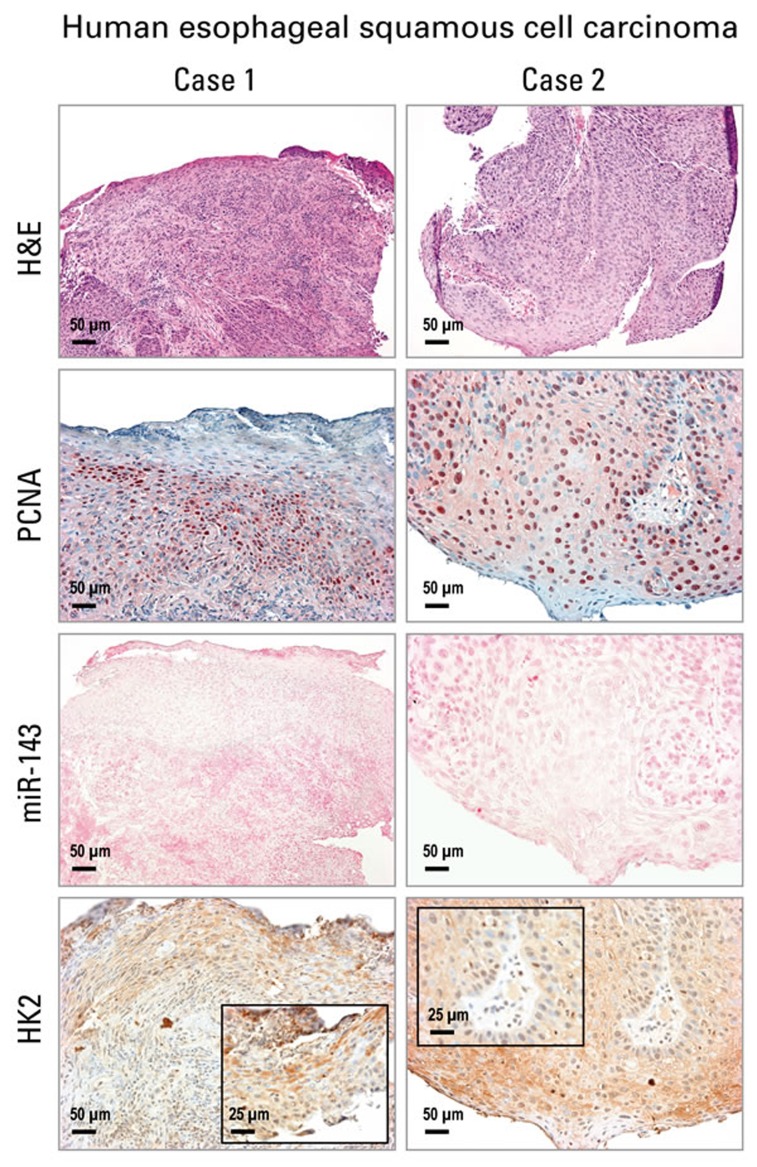
Analysis of cell proliferation, miR-143 and HK2 expression in human esophageal squamous cell carcinoma (ESCC) tissue by *in situ* hybridization and immunohistochemistry Representative hematoxylin and eosin [H&E]-stained and PCNA-stained ESCC tissues (2 cases are shown). miR-143 ISH signal (blue, 4-nitro-blue tetrazolium and 5-brom-4-chloro-3′-indolylphosphate; counterstain, nuclear fast red) was absent in ESCC tumor area. HK2 expression (brown, 3,3′-diaminobenzidine tetrahydrochloride) was moderate to strong in near serial formalin-fixed, paraffin-embedded sections of ESCC tumor tissue (Case 1 & 2). Scale bars: 50 μm (x200 magnification), and 25 μm (x400 magnification, inset) (*n* = 12 cases).

Using the same human ESCC tissues in which we previously documented overexpression of miR-31, miR-21, miR-223 by ISH [28], Figure 4 shows these human ESCC tissues were also highly proliferative with numerous PCNA-positive nuclei (*n* = 12 cases). While miR-143 ISH signals were absent in human ESCC tissues, strong cytoplasmic HK2 protein expression was present (Figure 4). This result is typical of all 12 patients studied and is consistent with that in the ZD preneoplastic/cancerous esophageal tissues.

## DISCUSSION

In the current study, untargeted metabolomic profiling by GC-TOF MS showed that the highly proliferative ZD rat esophagus has a distinct cancer-associated metabolomic signature as compared with nonproliferative ZS counterpart (Table 1). Glucose down-regulation (153-fold, *P* < 0.001) was accompanied by up-regulation of lactic acid (1.7-fold, *P* < 0.001), a result indicating a high rate of aerobic glycolysis (the Warburg effect), a common feature of cancer cell metabolism. At the same time, almost all of the 44 metabolites that were up-regulated were intermediate precursors needed for amino acid metabolism/protein biosynthesis, purine & pyrimidine metabolism, or lipid biosynthesis. These finding emphasize the requirement of cell proliferation to provide building blocks to support *de novo* biosynthesis needed to produce new cells [48]. Importantly, the dysregulated metabolites, including glucose, were highly Zn-responsive. Zn-replenishment rapidly increased glucose content and restored many deregulated metabolites to control levels (Table 2), accompanied by reversal of the hyperplastic phenotype. Taken together, the metabolomic data establish for the first time that the nutrient, Zn, regulates cancer metabolism in esophageal neoplasia development and reversal. This result is consistent with the ZR effect on the reversal of altered gene expression in esophageal neoplasia [16, 17].

To date, there are only a few metabolomic profiling studies in human ESCC; most of which were performed on biofluids from ESCC patients (plasma, serum, and urine). Only one study was conducted on human ESCC tissues *vs* normal mucosae using nuclear magnetic resonance, where glucose down-regulation and up-regulation of short-chain fatty acid were correlated with the stage of ESCC [57], consistent with our metabolomic profile of the preneoplastic rat esophagus induced by ZD.

Human cancers have been extensively profiled by transcriptomic- and miRNA-based studies and increasingly by metabolic profiling, owing to recent advances in metabolomic platforms. Efforts to integrate these multi-level events to form gene-metabolite networks have led to a better understanding of the molecular basis of cancer. In the current study, to decipher the mechanism underlying aberrant glucose metabolism induced by dietary ZD in the earliest stage of esophageal carcinogenesis, we performed an integrated analysis of metabolomics with our published transcriptomics for preneoplastic ZD rat esophagus [16]. This analysis uncovers a direct link between glucose down-regulation and up-regulation of the glycolytic enzyme HK2 (Figure 2). HK2 catalyzes the first step of glycolysis and is overexpressed in many human cancers, including ESCC. Several other enzymes in glycolysis are also increased in cancer cells, but unlike HK2 they are also expressed in normal cells [58]. Genetic deletion of HK2 has been reported to reduce the overall tumor burden in lungs in a genetic mouse lung cancer model, indicating a role of HK2 in tumor initiation [58]. In patients with ESCC, a positive correlation is well established between a high ^18^F-FDG uptake (a marker for tissue uptake of glucose) and HK2 activity in resected specimen [59, 60]. Thus, our finding of a HK2-glucose link in hyperplastic ZD esophagus highlights the critical role of HK2 in deregulated glucose metabolism in early esophageal tumor development by ZD.

It is now well recognized that miRNAs play a critical role in regulating cellular metabolism in health and disease, including cancer, thereby generating a bidirectional functional link in the crosstalk between miRNA and metabolism [34, 61]. The most pronounced metabolic alteration in cancer cells is deregulated uptake of glucose and associated aerobic glycolysis [14, 32]. Critical to cancer aerobic glycolysis is the glycolytic enzyme HK2 that catalyzes the first committed step in glycolysis [54]. In this study, integrated analysis of -omics datasets with miRNA expression profiling revealed a miR-143-HK2-glucose link in hyperplastic ZD esophagus. Moreover, we showed that miR-143 regulates cancer glycolysis in ZD-induced esophageal neoplasia by targeting HK2 (Figure 3), a result consistent with reports for lung cancer, renal cell carcinoma, head and neck squamous cell carcinoma, breast cancer, and prostate cancer [36-38, 62, 63].

A limitation of this current study is the fact that only the *HK2*-glucose gene-metabolite network is studied in detail. Future studies are needed to investigate other gene-metabolite connections in our integrated analysis of metabolomics and transcriptomics (Figure 2, Supplementary Figure 1). In addition, future studies are needed to unravel the mechanism(s) whereby dietary ZD regulates the expression of miR-143.

In summary, our metabolomic profiling reveals that increased consumption of glucose is the most prominent metabolic alteration in the highly proliferative ZD esophagus. The finding that ZR rapidly corrects aberrant metabolite levels, including that of glucose, indicates that Zn regulates glucose metabolism in esophageal cancer initiation and reversal. Integration of metabolomics with our published transcriptomics and microRNA profiling data revealed a miR-143-HK2-glucose pathway that underlies esophageal neoplasia by ZD. The finding shows that to support cell proliferation, ZD reprograms glucose metabolism by modulating the expression of miR-143, which targets HK2. Altogether, our data advance the understanding of the mechanistic role of Zn in ESCC development and prevention.

## MATERIALS AND METHODS

### Rat studies

The Animal protocol was approved by the Thomas Jefferson University Animal Care and Use Committee. Male weanling Sprague-Dawley rats (50 ± 5 g) were obtained from Taconic Laboratory (Germantown, NY). Custom-formulated ZD and ZS diets (Harlan Teklad, Madison, WI) were identical except for the amount of zinc, which was 3-4 ppm for ZD and ∼60 ppm for ZS diet. Weanling male rats (*n* = 48) were fed a ZD (*n* = 32) or ZS (*n* = 16) diet for 6 weeks to establish esophageal proliferation in ZD rats [16, 20, 27]. ZD rats were fed *ad libitum* and ZS rats were pair-fed to ZD animals to match the decreased food consumption of ZD rats [16, 20, 27]. After 6 weeks ZD rats evidenced increased cell proliferation in the esophagus, as assessed by increased expression of proliferating cell nuclear antigen PCNA [15]. Zinc gluconate (1.0 mg elemental zinc) in saline was then administered intragastrically to 16 ZD rats, which were immediately switched to the ZS diet to form the ZR group. At 72 hours after replenishment, all animals (ZD, ZR, and ZS, *n* = 16 rats/group) were killed. Whole esophagus was excised and collected onto ice to minimize further metabolism. Esophageal epithelia (*n* = 10 rats/group) were prepared by using a blade to remove the submucosal and muscularis layers, snap-frozen in liquid nitrogen, and stored at -80^o^C [16, 64]. The remaining esophageal samples were fixed in 10% buffered formalin and paraffin embedded.

### GC TOF MS data acquisition and processing

For metabolomic profiling, frozen esophageal samples from ZD, ZS, and ZR rats (*n* = 8 rats per group; 20 mg per esophagus) were shipped on dry ice to the NIH West Coast Metabolomics Center, University of California, Davis. Esophageal tissue was extracted and derivatized as described [40, 42]. For primary metabolites analysis by GC-TOF MS, the cold injection/automatic liner exchange gas chromatography-time of flight mass spectrometry (CIS-ALEX GC-TOF MS, Leco Pegasus IV) was employed using chromatographic and mass spectrometric parameters as previously described [40, 42]. From around 800 individual peaks detected per chromatogram, 353 genuine metabolites remained after extensive cleanup and filtering through the BinBase metabolomic database. Using the Fiehnlib libraries of over 1,200 mass spectra and retention indices for identified metabolites, 155 compounds per studies were be structurally identified by matching mass spectra and retention indices to authentic standards (MSI [65] level 1 identifications) or annotated by very high mass spectral similarities to the NIST14 library and close retention time predictions (MSI level 2 identifications, name followed by the label “NIST” in the Tables 1 and 2). A quality control sample for extracts was prepared by mixing small biofluid (∼5 μl) of each sample in a study set, thus providing a sample with the true representation of the breadth of metabolites present in the sample set.

### Statistical analysis

For analysis involving only 2 sets of data, a student’s *t*-test or welch *t*-test was used depending on a F-test for differences in variance. For analysis involving multiple comparisons of all 3 groups (ZD, ZS and ZR), a Shapiro-Wilk Normality test was performed for each group. If any group failed the normality test, an overall Kruskal-Wallis chi-squared test was performed to test if there were any differences among the groups. A significant Kruskal-Wallis result was followed by pairwise Wilcoxon rank sum test to identify the differences between individual groups. In the case where no group failed the normality test, the Levene’s homogeneity of variance test was performed. For datasets that were homoscedastic, a standard one-way ANOVA for overall differences was performed, followed by the Tukey HSD pot-hoc *t*-test for determination of differences among the groups. For heteroscedastic data, a Welch one-way ANOVA was performed, followed by the Games-Howell post hoc *t*-test for determination of differences among the groups. Statistical tests were 2-sided and were considered significant at *P*≤0.05. Statistical analysis was performed using R (http://www.R-project.org).

### Metabolome network visualization

Network analysis was used to investigate statistical and multivariate modeling results within a biochemical context. A biochemical and chemical similarity network [45] was calculated for all measured metabolites with KEGG [46] and PubChem CIDs [47]. Enzymatic interactions were determined based on product-precursor relationships defined in the KEGG RPAIR database. Molecules not directly participating in biochemical transformations, but sharing many structural properties, based on PubChem Substructure Fingerprints [66], were connected at a threshold of Tanimoto similarity coefficient ≥ 0.7.

The table of metabolites (nodes) and network properties (p-values, direction of change) provided by MetaMapp were imported into the Cytoscape software and networks were generated by the organic layout algorithm in Cytoscape. Red and blue nodes show the increase and decrease in the amounts of metabolites, respectively. The blue lines reflect chemical similarity and red lines show biochemical reactions. Labels are not shown for metabolites that did not pass significance tests.

### Integrating metabolomics with gene expression data

A genomics/metabolomics integration network from the data the metabolic profile in ZD rat esophagus was integrated with our published transcriptome dataset for this tissue ( > 2300 dysregulated genes, identical study design as metabolomics data) [16]. The network contains genes, proteins and metabolites. It was queried from significant genes and metabolites (*P-*value < 0.05) and mapped with log2 fold-change.

### Integrating gene expression data with miRNA profiling data

To find known miRNAs that regulate HK2, we searched our published miRNA profiling data for ZD esophagus [28].

### Rat esophageal samples for qPCR, TaqMan miRNA assay, ISH & IHC assays

For analyses involving 6 wks-ZD esophagus, esophageal tissues from the current metabolomic profiling study were used. For analyses involving esophageal tumor endpoint, archived samples of ESCC-bearing ZD esophagus and tumor-free control ZS esophagus from a previous study [28] were employed.

### RNA isolation

Esophageal epithelial samples frozen in liquid nitrogen were pulverized to a fine powder using a chilled hammer. Total RNA was extracted from the pulverized samples using an animal tissue RNA extraction Kit (#25700, Norgen Biotek, Ontario, Canada). RNA concentration of each sample was determined using a NanoDrop 1000 (Thermo Scientific). All RNA samples displayed a 260:280 ratio > 1.8, and a 260:230 ratio > 1.8.

### qPCR

cDNA was reverse transcribed using the High-Capacity cDNA Archive Kit (Applied Biosystems, Foster City, CA) according to the manufacturer’s protocol. qPCR was performed using pre-designed probes (Applied Biosystems), Psmb6 and Oaz1 as the normalizers, and the comparative Ct method.

### TaqMan miRNA assay

Reverse transcription of miRNAs was performed according to the manufacturer’s instructions (Applied Biosystems, Foster City, CA) with a reaction volume of 15 μl containing 350 ng of total RNA. Real-time qPCR was performed using StepOnePlus Real-time System (Applied Biosystems). Each miRNA and endogenous control (snoRNA and U87) was measured in triplicates. As an overall quality control, Ct (cycle threshold) values above 35 were excluded from analysis.

### Human ESCC samples

Twelve cases of FFPE human ESCC samples were obtained from Thomas Jefferson University Hospital (Philadelphia, PA).

### *In situ* hybridization

miRCURY locked nucleic acid (LNA)™ microRNA detection probes, namely, hsa-miR-143, rno-miR-31, and negative controls were purchased from Exiqon (Vedbaek, Denmark). The oligonucleotides are double DIG-labeled at the 5’- and 3’-ends. ISH was performed on 6 µm FFPE sections as previously described [26-28]. Following deparaffinization, rehydration in graded alcohol and proteinase K treatment, tissue sections were hybridized with miR-143 probe (20 nM), in hybridization buffer (Exiqon) at 57°C for 14 h in a hybridizer (Dako, Glostrup, Denmark). Following stringent washes in SSC buffers, the sections were blocked against unspecific binding of the detecting antibody, using DIG wash and blocking reagent. miRNA was localized by incubation with 4-nitro-blue tetrazolium (NBT) and 5-brom-4-chloro-3′-indolylphosphate (BCIP) (Roche, Mannheim, Germany). Nuclear fast red (Vector Lab., Burlingname, CA) was used as a counterstain.

### Immunohistochemistry (IHC)

FFPE tissues were deparaffinized, and rehydrated in graded alcohols. IHC was carried out as previously described [20, 27], using anti-hexokinase 2 antibody [3D3] (#NBP1-51643, mouse monoclonal, Novus Biologicals, Littleton, CO, USA) and PCNA antibody (clone PC-10, Ab-1, Thermo Scientific, Waltham, MA, USA), after citrate-based antigen retrieval. Protein was localized by incubation with 3-amino-9-ethylcarbazole substrate-chromogen (Dako, Carpinteria, CA, USA) or 3,3’-diaminobenzidine tetrahydrochloride (Sigma-Aldrich, St Louis, MO, USA).

### Microscopy

IHC and ISH analyses were performed by light microscopy using an Olympus BX51 microscope and photographs taken with a Spot RT3 camera and Spot software v. 4.6.

### Zn content analysis

Serum Zn content is determined by atomic absorption spectrometry [16].

## SUPPLEMENTARY MATERIALS FIGURE


