# Supplementary figures and images for: Integration of metabolomics, transcriptomics, and microRNA expression profiling reveals a miR-143-HK2-glucose network underlying zinc-deficiency-associated esophageal neoplasia
Source: Oncotarget. 2017 Jun 9;8(47):81910–25. doi: 10.18632/oncotarget.18434 (PMC5669858; doi:10.18632/oncotarget.18434)

## Supplementary Material

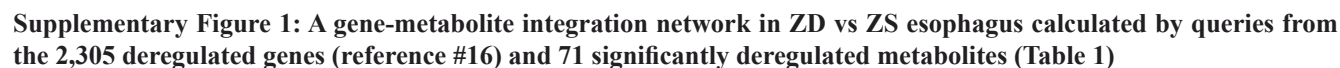

Supplement: Supplementary file 1 [file oncotarget-08-81910-s001.pdf]
